# The Triterpenoid CDDO-Me Inhibits Bleomycin-Induced Lung Inflammation and Fibrosis

**DOI:** 10.1371/journal.pone.0063798

**Published:** 2013-05-31

**Authors:** Ajit A. Kulkarni, Thomas H. Thatcher, Hsi-Min Hsiao, Keith C. Olsen, Robert Matthew Kottmann, Jason Morrissette, Terry W. Wright, Richard P. Phipps, Patricia J. Sime

**Affiliations:** 1 The Division of Pulmonary and Critical Care Medicine, Department of Medicine, University of Rochester, Rochester, New York, United States of America; 2 Lung Biology and Disease Program, University of Rochester, Rochester, New York, United States of America; 3 Department of Pathology, University of Rochester, Rochester, New York, United States of America; 4 Department of Microbiology and Immunology, University of Rochester, Rochester, New York, United States of America; 5 Department of Biostatistics and Computational Biology, University of Rochester, Rochester, New York, United States of America; 6 Department of Pediatrics, University of Rochester, Rochester, New York, United States of America; 7 Department of Environmental Medicine, University of Rochester, Rochester New York, United States of America; French National Centre for Scientific Research, France

## Abstract

Pulmonary Fibrosis (PF) is a devastating progressive disease in which normal lung structure and function is compromised by scarring. Lung fibrosis can be caused by thoracic radiation, injury from chemotherapy and systemic diseases such as rheumatoid arthritis that involve inflammatory responses. CDDO-Me (Methyl 2-cyano-3,12-dioxooleana-1,9(11)dien-28-oate, Bardoxolone methyl) is a novel triterpenoid with anti-fibrotic and anti-inflammatory properties as shown by our *in vitro* studies. Based on this evidence, we hypothesized that CDDO-Me would reduce lung inflammation, fibrosis and lung function impairment in a bleomycin model of lung injury and fibrosis. To test this hypothesis, mice received bleomycin via oropharyngeal aspiration (OA) on day zero and CDDO-Me during the inflammatory phase from days -1 to 9 every other day. Bronchoalveolar lavage fluid (BALF) and lung tissue were harvested on day 7 to evaluate inflammation, while fibrosis and lung function were evaluated on day 21. On day 7, CDDO-Me reduced total BALF protein by 50%, alveolar macrophage infiltration by 40%, neutrophil infiltration by 90% (p≤0.01), inhibited production of the inflammatory cytokines KC and IL-6 by over 90% (p≤0.001), and excess production of the pro-fibrotic cytokine TGFβ by 50%. CDDO-Me also inhibited α-smooth muscle actin and fibronectin mRNA by 50% (p≤0.05). On day 21, CDDO-Me treatment reduced histological fibrosis, collagen deposition and αSMA production. Lung function was significantly improved at day 21 by treatment with CDDO-Me, as demonstrated by respiratory rate and dynamic compliance. These new findings reveal that CDDO-Me exhibits potent anti-fibrotic and anti-inflammatory properties *in vivo*. CDDO-Me is a potential new class of drugs to arrest inflammation and ameliorate fibrosis in patients who are predisposed to lung injury and fibrosis incited by cancer treatments (e.g. chemotherapy and radiation) and by systemic autoimmune diseases.

## Introduction

Pulmonary Fibrosis (PF) is a devastating progressive disease in which normal lung structure and function are compromised by scarring. One hallmark of the fibrotic lung is excess deposition of extracellular matrix (ECM) proteins in areas that are enriched in proliferating fibroblasts and myofibroblasts. Myofibroblasts are characterized by expression of α-smooth muscle actin (αSMA) and calponin and produce many ECM proteins including collagen and fibronectin. Deposition of ECM proteins leads to impairment of normal lung function and structure, which compromises gaseous exchange and can lead to respiratory failure [1], [2]. PF can be caused by systemic diseases (such as rheumatoid arthritis and sarcoidosis), exposure to environmental agents (asbestos, silica), chemicals (chemotherapy drugs including bleomycin, busulfan, carmustine and chlorambucil), or radiation therapy [1], [3]. Idiopathic pulmonary fibrosis (IPF), in which the cause is unknown, is the worst form of lung scarring, with a median survival time of 2.9 years and no effective treatments [1].

Pulmonary fibrosis is a fatal complication of chemotherapy and thoracic radiation. Five to 40% of cancer patients develop drug-induced pulmonary injury, inflammation and fibrosis, resulting in significant morbidity. Mortality rates range from 2%–80% of cases, depending on the inciting agent [4]. Because the risk of pulmonary injury rises with cumulative dose of drugs or radiation, the risk of injury limits the use of otherwise effective therapies [3], [5]. While PF associated with some diseases–such as bronchiolitis obliterans organizing pneumonia (BOOP) and sarcoidosis–can be treated with steroids, other forms of PF due to chemo- and radiotherapy, including IPF fibrosis, can not be effectively treated. Current therapies only relieve symptoms and do not alter the course of the disease [1], [6]. However, unlike IPF, in the case of drug or radiation induced fibrosis, the initiation time of the disease is known. Therefore, there is an unmet need for effective ant-inflammatory and antifibrotic therapies, both to treat currently untreatable disease and for prophylactic use with cancer therapies to increase the drug dose and lower risk of lung toxicity.

2-cyano-3,12-dioxooleana-1,9-dien-28-oic acid (CDDO) is a novel therapeutic, which has potent anti-inflammatory and anti-neoplastic properties. For example, it blunts the NF-κB pro-inflammatory pathway and activates the Keap1-Nrf2 anti-oxidant pathway *in vitro* [7], and attenuates the response to LPS challenge *in vivo* [8]. We have reported that CDDO has potent *in vitro* anti-fibrotic activities in primary human lung fibroblasts (HLFs) from both normal [9]–[11] and IPF donors [10]. CDDO inhibits myofibroblast differentiation and expression of ECM proteins *in vitro*, by binding to other cellular proteins, such as transcription factors and signaling molecules, altering their activity [7].

Several derivatives of CDDO that have improved potency, bioavailability and stability are in preclinical development [7]. A more stable and orally available derivative of CDDO, methyl ester of CDDO (CDDO-Me), has been evaluated for lymphomas and diabetic kidney disease, which display inflammatory and fibrotic components [7]. An important knowledge gap is whether CDDO-Me also exhibits its anti-inflammatory and anti-fibrotic properties *in vivo.* Here, we test the concept that CDDO-Me will exhibit anti-inflammatory and anti-fibrotic properties in the bleomycin model of pulmonary fibrosis. Our data indicate that CDDO-Me has high translational potential as a pulmonary anti-inflammatory and anti-fibrotic therapy.

## Materials and Methods

### Ethics Statement

All animal procedures were approved and supervised by the University of Rochester University Committee on Animal Resources (UCAR permit number 2004-335R). Prior to surgical isolation of the lungs for analysis, mice were anesthetized with an I.P. injection of avertin (2,2,2-tribromoethanol, 250 mg/kg), followed by exsanguination; all efforts were made to minimize suffering. Primary human lung fibroblasts were derived from anatomically normal areas of lung tissues obtained from patients undergoing wedge biopsy for other medical reasons and excess tissue was obtained. All tissue donors provided informed written consent as described in study protocols approved by the University of Rochester Institutional Review Board and conforming to the Helsinki Declarations.

### Animals

Male C57BL/6J mice age 8–10 weeks were obtained from The Jackson Laboratory (Bar Harbor, ME) and housed at the University of Rochester. The mice were handled and maintained using microisolation techniques with daily veterinarian monitoring. All experiments were conducted under a protocol approved by the institutional animal care use committee of the University of Rochester Medical Center. The number of mice used for each analysis is shown in the figure legends.

### Inflammation model

Bleomycin (Teva Pharmaceuticals, Israel) was diluted in PBS and 2 U/kg was administered in a volume of 40 µl by oropharyngeal aspiration (OA) on day 0. Age-matched C57BL/6J mice were used as controls and given 40 μl PBS. Methyl 2-cyano-3,12-dioxooleana-1,9(11)dien-28-oate (CDDO-Me) was obtained from Reata Pharmaceuticals, (Dallas, TX) and dissolved in DMSO at a concentration of 10 mM. This stock was aliquoted and kept frozen at −80°C until use. The CDDO-Me stock was diluted in sterile normal saline immediately prior to use (final DMSO concentration was 0.1%). The treatment group received 400 ng of CDDO-Me in 40 µl by OA every other day beginning on day -1 (days -1, 1, 3 and 5). Vehicle control mice received 0.1% DMSO in saline. Experiments were performed with n = 6 mice per group with one independent experiment.

Inflammation was assessed on day 7 by isolating bronchoalveolar lavage (BAL) fluid for differential cell count and protein measurement. Briefly, mice were anesthetized with 250 mg/Kg i.p. Avertin (2,2,2-tribromoethanol, Sigma) and euthanized by exsanguination. The lungs were removed and lavaged twice with 0.5 ml of phosphate-buffered saline (PBS). The lavage fluid was centrifuged, the total BAL cell number was determined by hemacytometer and differential cell counts were performed on Cytospin-prepared slides (Thermo Shandon, Pittsburgh, PA) stained with Richard Allen three-step stain (Thermo Fisher Scientific Inc., Pittsburgh, PA). Left lungs were frozen immediately for later biochemical analysis. A lobe of the right lung was stored in RNAlater (Qiagen, Valencia, CA) for isolation of mRNA and remaining right lung lobes were flash frozen in liquid nitrogen for the analysis of hydroxyproline or SDS-PAGE.

### Fibrosis model

After performing the inflammation studies, we obtained a new lot of bleomycin from a different supplier (Hospira, Lake Forest, IL) and determined through a pilot experiment that the bleomycin dose needed to be reduced. Consequently the fibrosis experiments were performed using 1.5U/Kg of bleomycin. With the reduction in bleomycin dose we also reduced the amount of CDDO-Me used. Two independent experiments were performed (n = 8–10 mice per group in each experiment) using 300 ng CDDO-Me in one experiment and 350 ng CDDO-Me in the other. To assess the results, a general linear model employing a two-way analysis of variance with interaction was developed to evaluate the effect of treatment and CDDO-Me dosage (see the supplemental material for details of the statistical analysis). Mice received 1.5U/kg of bleomycin in a 40 µl volume of PBS by OA on day 0, and were treated with either 300 ng or 350 ng CDDO-Me in 40 µl volume by OA every other day from day -1 to day 9. Control groups received bleomycin plus vehicle.

Fibrosis was assessed on day 21. The mice were anesthetized and euthanized as described. The lungs were removed without lavaging. The left lung was inflated in 10% neutral buffered formalin at 25 cm water pressure, and used for histology. A lobe of the right lung was stored in RNAlater (Qiagen, Valencia, CA) for isolation of mRNA and remaining right lung lobes were flash frozen in liquid nitrogen for the analysis of hydroxyproline or SDS-PAGE.

### ELISA

Inflammatory cytokines were measured in lung homogenates as described [12]. Briefly, lungs were homogenized in Buffer A (10 mM HEPES, pH 7.9, 10 mM KCL, 0.1 mM EDTA, and 1 mM DTT), centrifuged, and the clarified homogenate was assayed for IL-6, KC and TGFβ using commercial ELISA kits according to the manufacturer's directions (R&D Systems, Minneapolis MN). To measure total TGFβ, samples were acid activated as directed by the manufacturer to release latent TGFβ in the sample. Cytokine values were normalized to total protein in the homogenate as determined by the bicinchoninic acid (BCA) assay (Pierce, Rockford, IL) and expressed as pg cytokine/mg total protein.

### Hydroxyproline assay

Hydroxyproline content was measured in the right lung using the modified Woessner method as previously reported [13]. Lung tissue was homogenized in water and protein was precipitated using 10% TCA. Samples were then hydrolyzed overnight in 6 N HCL at 110°C. Samples were neutralized with the addition of NaOH, and chloramine T reagent solution was added to the samples for 20 minutes and then inactivated with 3.15 N perchloric acid. Ehrlich's solution was then added to the samples and they were incubated at 60°C for 20 minutes. A standard curve was prepared from purified hydroxyproline (Sigma-Aldrich, St. Louis, MO). Absorbance was read at 560 nM.

### Histology

Mice were euthanized and left lungs inflated at 25 mmHg H_2_O pressure with 10% neutral buffered formalin and incubated overnight in formalin for fixation. Lungs were dehydrated in 70% ethanol, processed using standard procedures and embedded in paraffin sections. Sections of 5 μm thickness were cut, mounted on slides, and stained with Gomori Trichrome (Richard Allen brand, Thermo Fisher Scientific Inc., Pittsburgh, PA) using the manufacturer's suggested protocol. The histology slides were scored for fibrosis by a blinded pathologist on the scale of 0 (minimum) to 4 (maximum) as described previously [14]. Immunohistochemistry was performed as described [13] using primary antibodies to αSMA (Sigma-Aldrich, St. Louis, MO). Antigen retrieval was performed using pH 6.0 citrate buffer in a 95^o^C water bath for 20 minutes. Slides were blocked with 1% goat serum in PBS and incubated with primary antibody overnight. The slides were washed and incubated with secondary antibody conjugated to biotin (Vector, Burlingame, CA) then streptavidin HRP (Jackson ImmunoResearch, West Grove, PA), and developed using Nova Red (Vector, Burlingame, CA) staining solution. Slides were then counterstained with hematoxylin (Richard Allen brand, Thermo Fisher Scientific Inc., Pittsburgh, PA) and mounted.

### RNA preparation and real-time PCR

Total lung tissues were immersed in RNAlater (Qiagen, Valencia, CA) at 4°C prior to processing using RNeasy, according to the manufacturer's protocol (Qiagen, Valencia, CA). RNA (1.0 μg) was incubated with PCR buffer, 0.5 μg of oligo (dT)12–18 primer (Life Technologies, Grand Island, NY), 10 mM deoxy-nucleotide-triphosphate (dNTP) for 10 minutes at 70°C and 5 minutes in ice water, followed by addition of 40 U of recombinant RNasin RNase inhibitor (Promega, Madison, WI), 0.1 mM DTT, and 200 U of Superscript III reverse transcriptase (RT; Life Technologies, Grand Island, NY). The mixture was further incubated for 5 minutes at room temperature, 60 minutes at 50°C, and 15 minutes at 70°C. The reaction contents were diluted to 80 μl volume and stored at −20°C. Negative controls contained no RT enzyme.

Quantitative real-time RT-PCR reactions were performed using a Bio-Rad iCycler with SYBR Green Supermix (Bio-Rad, Hercules, CA) according to the supplier's recommended protocol, with the following modifications. For amplification of the mouse collagen I (COL1A1), αSMA (ACTA2), fibronectin and 18S rRNA, the reactions contained 3 mM MgCl_2_ and 0.15 μM of each primer. Oligomer primers were obtained from Integrated DNA Technologies (Coralville, IA). Primer sequences were described previously [14] except for 18S sense: GCTTGCTCGCGCTTCCTTACCT and anti-sense: TCACTGTACCGGCCGTGCGTA.

### Western blots

Lung tissue was homogenized in ice cold PBS with protease and phosphatase inhibitor cocktail (Sigma-Aldrich, St. Louis, MO), diluted in 2x NP-40 lysis buffer supplemented with protease inhibitor, phosphatase inhibitor and 1 mM PMSF (Sigma-Aldrich, St. Louis, MO). Total protein (30–50 µg) were resolved by 10% SDS-PAGE, electrophoretically transferred to nitrocellulose membranes, and specific proteins were detected by standard Western blotting and chemiluminescence (Western Lightning, Perkin-Elmer, Wellesley, MA). Kodak Molecular Imaging Software (Rochester, NY) was used to perform densitometry on Western blot films and the band intensities were normalized to the loading control. The following primary antibodies were used: αSMA (Cat no: A2547, Sigma-Aldrich, St. Louis, MO), GAPDH (Cat no:ab8245, Abcam, Cambridge, MA) as primary and goat anti-mouse (Cat no:115-035-146, Jackson ImmunoResearch Laboratories, Inc., West Grove, PA) as the secondary antibody.

### Lung function testing

Lung compliance and respiratory rate were measured as described by us [15], [16]. Respiratory rate was measured using whole body unrestrained chambers (BUXCO Electronics Inc., Wilmington, NC) on live mice on day 20, one day before euthanasia and harvest. Compliance was determined immediately prior to euthanasia on day 21. Briefly, live ventilated mice were anesthetized and placed in a whole body plethysmograph (BUXCO Electronics Inc., Wilmington, NC) connected to a Harvard rodent ventilator (Harvard Apparatus, Southnatic, MA). Dynamic lung compliance was normalized to the peak body weight of the animal. Data was collected and analyzed using the Biosystems XA software package (BUXCO Electronics Inc., Wilmington, NC).

### Statistical analysis

Inflammation-related outcomes (7 day harvest) were assessed by one-way ANOVA using GraphPad Prism version 5. Because fibrosis was assessed in two independent experiments using different doses of CDDO-Me (300 ng and 350 ng), a general linear model employing a two-way analysis of variance with interaction was developed to assess the effect of treatment and CDDO dosage and analyzed using R 2.12.2 (The R Foundation for Statistical Computing, Vienna, Austria) on a Windows XP platform. Please see the Supporting Information for the details of the statistical analysis (Tables S1 to S4).

## Results

### CDDO-Me limits lung inflammation following bleomycin injury

We previously reported that CDDO has potent anti-fibrotic effects *in vitro* [9]–[11]. Several reports have indicated that the derivative CDDO-Me is 5-10-fold more potent than the parent compound and has increased bioavailability [17]–[20]. To verify these observations in primary human lung fibroblasts, we performed a comparison of CDDO-Me with CDDO. As CDDO-Me at 120 nM is at least as effective at inhibiting expression of αSMA (Figure S1A) and calponin (not shown), as 480 nM CDDO, we conclude that CDDO-Me is at least 4-fold more potent than CDDO in this in vitro assay. Even though CDDO-Me is a more potent inhibitor of myofibroblast differentiation, it has similar cytotoxicity to CDDO (Figure S1B).

**Figure 1:**
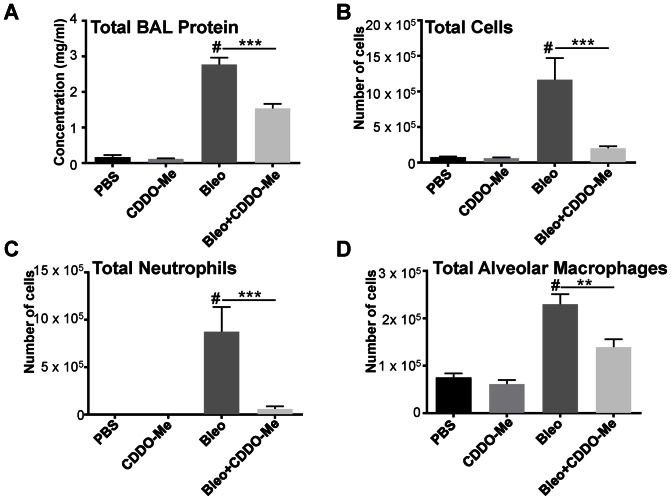
CDDO-Me reduces total BALF protein and cellular infiltration. Groups of mice (6 per group) were treated with bleomycin or PBS by inhalation on day 0, and with either CDDO-Me (400 ng/day) or control vehicle (Veh) by inhalation on days -1, 1, 3 and 5 and harvested on day 7. Lungs were lavaged and BALF was analyzed for total protein (**A**), total cell number (**B**), neutrophils (**C**) and alveolar macrophages (**D**). CDDO-Me potently reduces BALF protein, total cells, neutrophils and alveolar macrophages. These data are the mean ± SE of n = 6 mice per group (*p≤0.05, ** p≤0.01, compared to bleomycin+vehicle and #p≤0.01 compared to PBS+veh by one-way ANOVA).

Inhalation of bleomycin causes acute lung injury and inflammation that peaks around day 7 [21]. Therefore to evaluate the efficacy of CDDO-Me *in vivo* at reducing bleomycin-induced inflammation, groups of 6 mice received a single dose of bleomycin by OA and were treated with either vehicle or CDDO-Me on days -1, 1, 3 and 5. Mice were sacrificed on day 7 and BALF was isolated. Bleomycin alone increases BAL total protein, an indicator of edema and epithelial barrier breakdown. CDDO-Me reduced BAL total protein levels 50% (Fig. 1A). Bleomycin also promoted acute cellular inflammation as demonstrated by a strong influx of total inflammatory cells, alveolar macrophages and neutrophils (Fig. 1B–D). CDDO-Me potently and significantly reduced total bleomycin-induced cell infiltration in the BALF by 90%, neutrophils by 90% and BAL macrophages by 50%.

### CDDO-Me reduces inflammatory and fibrotic cytokine expression in lung homogenate

**Figure 2:**
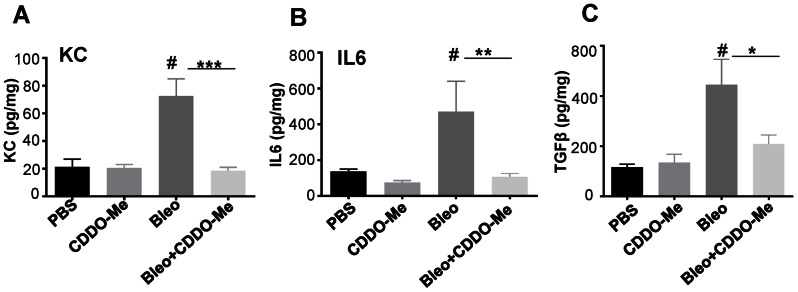
CDDO-Me inhibits production of IL6, KC and TGFβ in total lung homogenate. Mice were treated either with bleomycin or CDDO-Me as described and harvested on day 7. Cytokines in total lung homogenate were measured by ELISA and normalized to total protein. Bleomycin induces, and CDDO-Me significantly inhibits, KC (**A**), IL6 (**B**) and TGFβ (**C**). These data represent six independent animals and analyzed using one-way ANOVA (mean ± S.E. shown, *** p≤0.001, compared to bleomycin+vehicle and #p≤0.001 compared to PBS+veh).

Next, we examined key pro-inflammatory cytokines in lung homogenates as another marker of inflammation. IL-6 is involved in the pathogenesis of various inflammatory diseases and is also implicated in the pathogenesis of bleomycin-induced lung injury and subsequent fibrotic changes [22]. KC (CXCL1) is a key neutrophil chemokine in mice and is also upregulated in the bleomycin model [23]. CDDO-Me significantly reduced the expression of both KC and IL-6 by 90% (Fig 2A–B). We also measured TNF-α, but the levels were not elevated in the bleomycin group (data not shown), therefore we are unable to conclude whether CDDO-Me reduces the levels of TNF-α. We also examined the concentration of the pro-fibrotic cytokine TGFβ in the lung homogenates using ELISA. As expected, bleomycin treatment significantly upregulated total TGFβ, which was inhibited more than 50% by CDDO-Me (Fig. 2C).

### CDDO-Me reduces early upregulation of fibrotic gene expression

**Figure 3:**
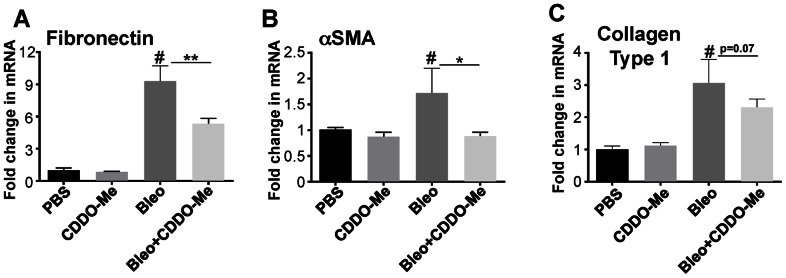
CDDO-Me decreases fibrotic gene expression. Mice were treated with bleomycin with or without CDDO-Me (as described in methods and materials). On day 7, the mice were euthanized and RNA prepared from a right lung lobe analyzed by RT-qPCR. Bleomycin significantly upregulates expression of fibronectin (**A**), αSMA (**B**) and type 1 collagen (**C**) at day 7. CDDO-Me significantly inhibited expression αSMA and FN. There was also a trend toward inhibition of collagen expression but this was not significant. These data represent six independent animals and analyzed using one-way ANOVA (mean ± S.E. shown, *p≤0.05, ** p≤0.01, compared to bleomycin+vehicle and #p≤0.01 compared to PBS+veh).

In the bleomycin model, lung fibrosis is strongly evident histologically by day 21. Upregulation of pro-fibrotic genes such as αSMA, collagen and fibronectin occurs as early as day 3 [21]. αSMA is a marker for myofibroblasts while collagen and fibronectin are ECM proteins secreted by fibroblasts and myofibroblasts. Here, mRNA levels for fibronectin, collagen I, and αSMA mRNA were all significantly increased 7 days after bleomycin exposure (Fig. 3). CDDO-Me inhibited 50% of the increase in fibronectin and αSMA RNA levels (Fig. 3A, B). CDDO-Me also reduced the increase in collagen mRNA by 30%, although this trend was not significant (p = 0.07) (Fig. 3C). These data show that CDDO-Me alters the early fibrotic events and decreases expression of pro-fibrotic genes.

### CDDO-Me inhibits the development of lung fibrosis after bleomycin challenge

**Figure 4:**
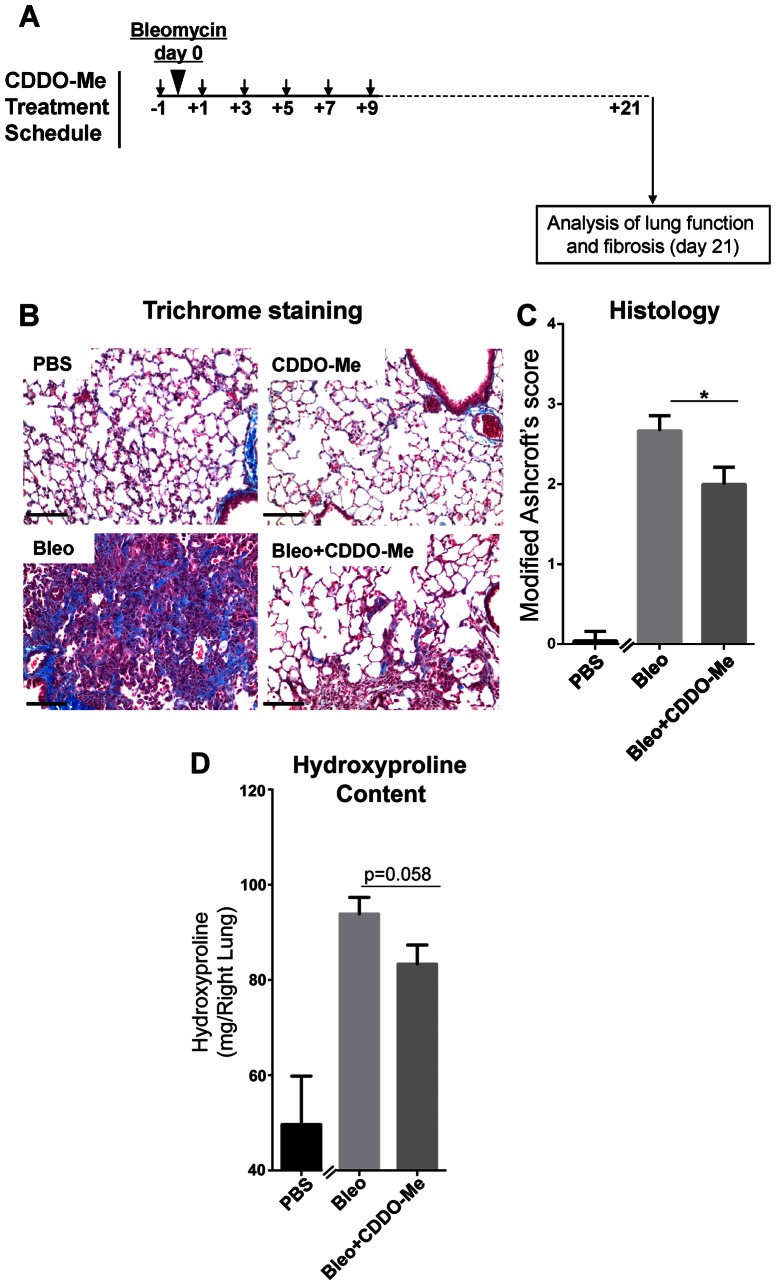
CDDO-Me reduces fibrosis in mice treated with bleomycin. Mice received bleomycin on day 0, and CDDO-Me or vehicle was given from day -1 to day 9 and lungs were harvested on day 21 (**A**). The left lungs were processed for histology and stained with Gomori's trichrome to visualize collagen deposition (blue) and overall fibrosis. Representative sections from each group are shown (**B**). Sections were scored by a blinded reviewer; CDDO-Me significantly reduced fibrosis score (**C**). Results shown are the mean± SEM for 14–16 mice per group and were analyzed using a two-sided t-test as described in methods and materials. A portion of the right lung was homogenized and hydroxyproline content was determined. Hydroxyproline contents increased in bleomycin-treated lungs and was reduced by CDDO-Me treatment (**D**) (mean ± SEM for n = 14–16, p = 0.0578 using two-sided t-test as described in the statistical supplement. Scale bar  = 100 μm.

Because CDDO-Me was effective at reducing early inflammation and expression of pro-fibrotic genes, we tested whether early treatment with CDDO-Me (days -1 to 9) would prevent late development of lung fibrosis (day 21). Mice received a single dose of bleomycin, and were treated with CDDO-Me using a pre-treatment strategy (day -1 to day 9) (Fig. 4A). Mice were euthanized on day 21 and fibrosis was evaluated by histochemical staining for collagen (Gomori's Trichrome method). CDDO-Me restricted deposition and overall distribution of collagen, a key outcome measure of fibrosis (Fig. 4B). Bleomycin-induced histological fibrosis was scored by a blinded pathologist and was significantly reduced by treatment with CDDO-Me (Fig. 4C). We also measured total lung hydroxyproline, a measure of collagen content. Bleomycin alone increased lung hydroxyproline content by 100%, this increase was reduced by 30% with CDDO-Me treatment, although the results were not quite significant (p = 0.058) (Fig. 4D). Taken together, these data indicate that early administration of CDDO-Me reduces fibrotic outcomes.

### CDDO-Me reduces expression of type I collagen and fibronectin

**Figure 5:**
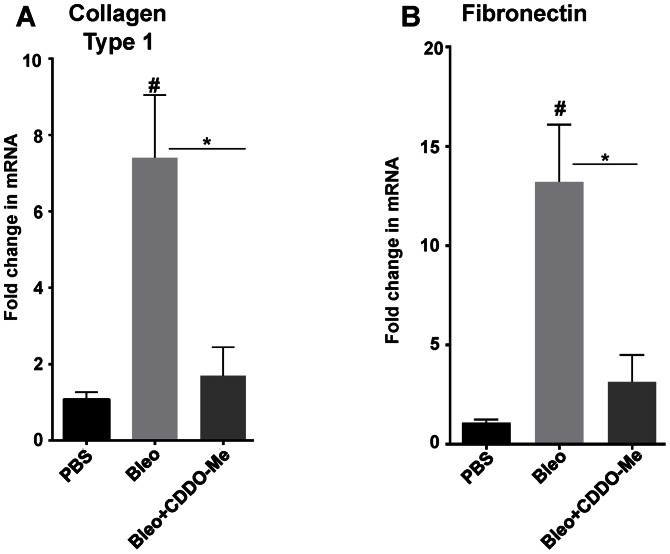
CDDO-Me inhibits expression of collagen I and fibronectin. Mice received bleomycin on day 0, and 350 ng of CDDO-Me or vehicle was given from day -1 to day 9 and lungs were harvested on day 21, a portion of right lung was used to measure mRNA and analyzed by RT-qPCR for collagen I (Col1A1) specific primers and normalized to 18S mRNA. CDDO-Me inhibited bleomycin–induced up-regulation of collagen I mRNA *p≤0.05 (**A**). CDDO-Me also reduced bleomycin–induced up-regulation of fibronectin expression, *p≤0.05 (**B**). These data represent 8–10 independent animals and analyzed using one-way ANOVA (mean ± S.E. shown, *p≤0.05, ** p≤0.01, compared to bleomycin+vehicle and #p≤0.01 compared to PBS+veh).

Since we noted a decrease in collagen I and fibronectin mRNA with CDDO-Me treatment at day 7 (Fig. 3C), we also evaluated their mRNA expression at day 21. As expected, mice treated with bleomycin alone had significantly higher collagen I expression than untreated mice (p≤0.01). We observed that CDDO-Me significantly inhibited the bleomycin-induced increase in collagen I (Fig. 5A). Consistent with our observation at day 7 (Fig. 3C), the expression of fibronectin was significantly higher in the bleomycin-treated mice as compared to PBS alone but was approximately 70% lower in CDDO-Me treated mice as compared to the bleomycin treated mice (Fig. 5B). These results indicate that early administration of CDDO-Me have long-lasting effects on the expression of fibrotic genes.

### CDDO-Me treatment reduces distribution and expression αSMA

**Figure 6:**
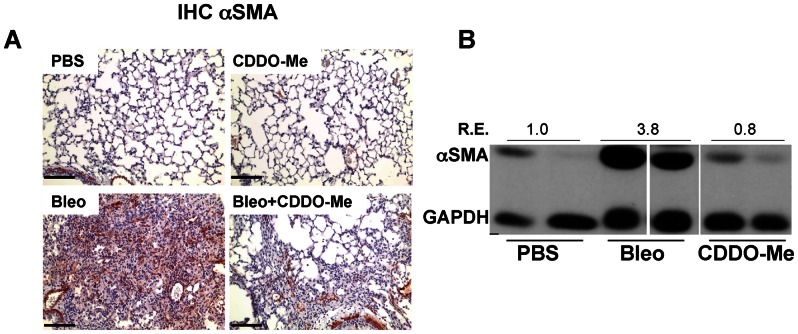
CDDO-Me reduces αSMA deposition and expression. Treatment of mice was carried out as described in Figure 4. Left lung sections processed for histological analysis and stained using an antibody against αSMA (brown) and counterstained with hematoxylin (blue). CDDO-Me reduces amount and distribution of αSMA positive cells in the treatment schedule. Scale bar  = 100 μm (**A**). Immunoblots were performed on total lung homogenate to detect expression of αSMA. CDDO-Me inhibited bleomycin–induced up-regulation of αSMA (**B**). Protein lysates from all the indicated samples were electrophoretically separated on the same gel, and representative lanes from a single experiment (n = 2) are shown here. Relative changes in the average expression of αSMA/GAPDH (R.E.) are as indicated in the figure for PBS, Bleo and Bleo+CDDO-Me.

αSMA is a marker for myofibroblast differentiation and is a sensitive indicator of the presence of fibrogenic cells in lung tissue. However, in contrast to collagen mRNA levels which remain elevated, consistent with the observations by Phan *et al.* [24] we observe that αSMA levels are transiently upregulated in this model, with higher levels on day 7 and 14 but little increased on day 21 (data not shown). Therefore, to assess myofibroblast differentiation and proliferation we measured αSMA protein by Western blot and immunohistochemistry. Bleomycin strongly induced expression of αSMA in mouse lungs at 21 days. The appearance of αSMA positive cells was reduced by treatment with CDDO-Me (Fig. 6). αSMA expression was quantified by Western blot analysis of whole lung homogenates. CDDO-Me dramatically and significantly reduced upregulation of αSMA by bleomycin.

### CDDO-Me improves lung functions after bleomycin challenge

**Figure 7:**
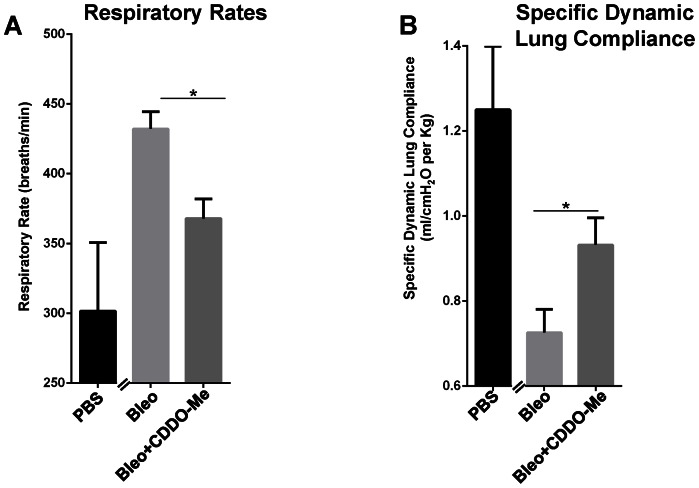
CDDO-Me improves lung functions of bleomycin treated mice. Mice (n = 13–16) were treated with bleomycin and CDDO-Me as described in Figure 4. (A) Respiratory rates (respirations/minute) were monitored on day 20 prior to harvesting the lungs on day 21.(B) Specific dynamic lung compliance was measured on day 21 immediately prior to euthanasia. (p≤0.05, using two-sided t-test as described in methods and materials).

We also assessed lung function in these animals. Lung function testing is the primary method of clinical diagnosis of fibrosis in patients, but is generally not performed in animal studies. Bleomycin increases respiratory rates due to lung scarring [25]. Respiratory rates of bleomycin and CDDO-Me treated mice were assessed non-invasively on day 20, one day prior to euthanasia. Mice treated with bleomycin alone had significantly higher respiratory rates than untreated mice (p≤0.05). Importantly, CDDO-Me potently and significantly lowered bleomycin-induced increases in respiratory rates (p≤0.01) (Fig. 7A). Decreased compliance is one of the cardinal clinical signs of lung fibrosis, as the accumulation of ECM proteins and scar tissue increases lung stiffness [25]–[27]. On day 21, immediately prior to euthanasia, the mice were immobilized and specific dynamic lung compliance was measured. Bleomycin alone induced a dramatic loss of lung compliance, which is partially and statistically significantly rescued by early treatment with CDDO-Me (p≤0.05) (Fig. 7B).

## Discussion

Therapy for inflammatory and scarring lung diseases is challenging [1]. While there is much attention on the disease IPF –where inflammation is not prominent, especially in later stages [1], [27] –there are many fibrotic diseases where inflammation is likely key in pathogenesis. For example, chemotherapy agents such as bleomycin, busulfan, carmustine and chlorambucil incite both inflammation and fibrosis [3], [4]. In addition, thoracic and whole body radiation used for the treatment of lymphoma, breast and lung cancer and for bone marrow transplantation causes dose-dependent fibrosis and pneumonitis in approximately 10% of patients [3]. Lung is the dose-limiting organ for bleomycin [28] and for radiation conditioning for bone marrow transplant [29]. The development of prophylactic treatment for therapy-induced lung fibrosis could benefit hundreds of thousands of patients per year, by preventing life-threatening disease and by relaxing constraints that limit the effectiveness of therapy.

CDDO-Me is a novel triterpenoid compound with anti-inflammatory, anti-proliferative and anti-neoplastic properties [7], [30]. We previously reported that the parent compound CDDO strongly inhibited TGFβ-induced myofibroblast differentiation of primary human lung fibroblasts [2], [5], [11]. We and others have reported that CDDO [31]–[33] and its derivatives [30], [34] are potent inhibitors of inflammation *in vivo* [34], [35] and *in vitro* [7], [36]. CDDO-Me was found efficacious in a phase 2 study [37] for diabetic kidney disease, which has inflammatory and fibrotic components. Here, we report that CDDO-Me inhibited bleomycin-induced lung inflammation and fibrosis in a mouse model when given during the inflammation phase. At day 7, the peak of the inflammatory phase, CDDO-Me significantly reduced cellular inflammation, pro-inflammatory and pro-fibrotic cytokines. Of particular note, CDDO-Me strongly suppresses TGFβ and IL-6. TGFβ is a key profibrotic cytokine that drives differentiation of lung fibroblasts to myofibroblasts [1], [38], [39]; TGFβ has also been shown to upregulate IL-17A which promotes fibrosis in multiple models [40], [41]. IL-6, in addition to being a broad pro-inflammatory marker, is a target for IL-1β-induced lung fibrosis [42]. Although we did not directly measure IL-1β, CDDO-Me has broad anti-inflammatory effect against multiple cytokines involved in fibrosis.

At day 21, early administration of CDDO-Me reduced late histological fibrosis and accumulation of collagen and prevented bleomycin-associated declines in lung function. Early administration of CDDO-Me also inhibited both early and late expression of ECM proteins including fibronectin. Fibronectin is a key effector in fibrosis as it is involved in cell adhesion, migration, and binding to integrins, collagen, and other matrix proteins, thus contributing to the overall fibrotic milieu. We observed that the repression of collagen mRNA (Fig. 5) is greater than the reduction of the total hydroxyproline contents of the lung (Fig. 4). It may be that the moderately elevated collagen mRNA levels are sufficient to lead to a larger increase in total hydroxyproline over time, or it may be that collagen mRNA levels vary on different time scales.

Bleomycin exposed fibrotic mouse lungs have low dynamic compliance due to thickened pulmonary parenchyma caused by scarring [25]. Mice treated with bleomycin also increase their respiratory rate to compensate for poor ventilation and gas exchange abnormalities. Not only did CDDO-Me improve biochemical and histological measures of fibrosis, it also significantly improved lung function. CDDO-Me significantly reduced the increase in respiratory rate and significantly improved compliance in bleomycin treated mice (Fig. 7). Our study underscores the utility of pulmonary function studies, which are usually not done in preclinical animal models. We show that CDDO-Me reduced markers of fibrosis and, importantly, it significantly improved overall lung function in this mouse model and ‘translates’ the observed antifibrotic and strong anti-inflammatory actions of CDDO-Me into clinically relevant measurements (Fig. 5). This is the first report that demonstrates the efficacy of bardoxolone methyl (CDDO-Me) as an anti-inflammatory, as well as antifibrotic drug, in the bleomycin mouse model *in vivo*. Taken together these data showed that early administration of CDDO-Me potently inhibited inflammation, reduced fibrotic outcomes and significantly improved lung function in bleomycin treated mice.

CDDO targets multiple anti-fibrotic pathways using *in vitro* systems. For example, CDDO-Me is a PPARγ ligand [43], and we [11] and others [44] have shown that activation of the PPARγ transcription factor inhibits differentiation of lung fibroblasts to myofibroblasts. Additionally, CDDO-Me is a potent electrophile and activates multiple cellular pathways via multiple PPARγ-independent mechanisms, including the activation of Keap1-Nrf2 pathway and inhibition of the PI3K-Akt pathway [45]. Nrf2 is a master regulator of the antioxidant response and, by inducing oxidative stress response genes, CDDO-Me may reduce the initial bleomycin injury. PI3K-Akt is a pro-survival and anti-apoptosis factor that is activated in fibrosis and inhibited by CDDO [10]. We do not yet know which of these mechanisms are most important *in vivo*, but it is interesting that bleomycin and thoracic radiation both induce DNA damage, oxidative stress and apoptosis [46], which implicates the Keap1-Nrf2 and Akt pathways. Interestingly, CDDO-Me reduced levels of total TGFβ in the lungs of bleomycin-treated mice at day 7, although whether this is related to its anti-inflammatory properties or one of its anti-fibrotic effects is unknown.

The risk of lung fibrosis from chemotherapy and thoracic radiation is dose-dependent, a factor that influences therapeutic planning and limits the potential usefulness of these treatments. Following chemotherapy between 5–40% of patients develop lung injury and fibrosis depending on the dose and the drug [15]. It important to human health to develop a prophylactic treatment that could be given at the same time as the therapy, or shortly thereafter, that would reduce the risk of developing fibrosis. Here, it is especially worth noting that CDDO-Me given only during the initial inflammation phase not only inhibited early injury, but also later development of fibrosis, and that reductions in fibrotic gene expression and proteins (fibronectin, type 1 collagen and αSMA) were persistent, seen at day 21, i.e. 12 days after last drug treatment.

This present study does not address the possibility of using CDDO-Me to treat established fibrosis such as IPF. We did carry out an initial study using a late treatment model (beginning on day 9) and found a trend toward reduced fibrosis that was not statistically significant (data not shown). While it may be that we only need to optimize the dose, timing or other variables, it is worth noting that early inflammation is a significant component of the bleomycin model that complicates efforts to confirm an anti-fibrotic role for CDDO-Me in the absence of inflammation. We suggest that future animal studies should use a less inflammatory lung fibrosis model, such as transient overexpression of TGFβ [47]. However, because CDDO-Me is effective in preventing bleomycin-induced fibrosis when given at the early inflammatory stage, we suggest it may be useful in lung fibrotic diseases with more classical inflammatory component, such as sarcoidosis or rheumatoid arthritis.

Based on our studies, prophylactic treatment with CDDO-Me is a novel strategy in drug-induced ILD, as well as in radiation-induced lung disease, which have major inflammatory components. If we can mitigate the side effects of these useful therapies, patients can continue to receive the life-saving treatments longer and at higher dose. It is also important to note that CDDO-Me was effective when given by inhalation, which may limit systemic side effects. We believe CDDO-Me has high translational potential as a human therapy to arrest the cellular pathology underlying lung inflammation and fibrosis, and we expect that CDDO-Me or its derivatives can be rapidly translated to human subjects who are predisposed to lung injury, inflammation and fibrosis.

## Supporting Information

Figure S1 **CDDO and CDDO-Me inhibit TGFβ-induced αSMA in a dose dependent manner.** (**A**) Primary HLFs were grown until 70–80% confluent, serum starved for 24 hours and treated with the indicated concentrations of CDDO or CDDO-Me for 48 hours. Total cell lysates were prepared, and subjected to SDS-PAGE followed by immunoblotting. The blot was probed with antibodies against αSMA and loading control GAPDH. (**B**) Primary HLFs were treated with TGFβ alone, in combination with different doses of either CDDO or CDDO-Me for 72 hours with indicated concentrations or left untreated, and LDH release was measured (nmol/min/mL). (n = 3, mean ± S.E. shown, groups were not significantly (n.s.) different from one another as measured by one way ANOVA). These data indicate that LDH release does not increase in response to either CDDO or CDDO-Me.
(TIF)

Table S1 **Preliminary analysis of the four control groups.**
(DOCX)

Table S2 **Tests for the significance of the interaction term in the full model.**
(DOCX)

Table S3 **Adjusted mean responses for the one treatment strategy and one positive control group.**
(DOCX)

Table S4 **Test for the treatment strategy versus the positive control.**
(DOCX)

## References

[1] Kottmann RM, Hogan CM, Phipps RP, Sime PJ (2009) Determinants of initiation and progression of idiopathic pulmonary fibrosis. Respirology 14: 917–933. 19740254 10.1111/j.1440-1843.2009.01624.x PMC3884519

[2] Nathan SD, Noble PW, Tuder RM (2007) Idiopathic pulmonary fibrosis and pulmonary hypertension: connecting the dots. Am J Respir Crit Care Med 175: 875–880. 17255562 10.1164/rccm.200608-1153CC

[3] Camus P, Fanton A, Bonniaud P, Camus C, Foucher P (2004) Interstitial lung disease induced by drugs and radiation. Respiration 71: 301–326. 15316202 10.1159/000079633

[4] Limper AH (2004) Chemotherapy-induced lung disease. Clin Chest Med 25: 53–64. 15062597 10.1016/S0272-5231(03)00123-0

[5] Dimopoulou I, Bamias A, Lyberopoulos P, Dimopoulos MA (2006) Pulmonary toxicity from novel antineoplastic agents. Ann Oncol 17: 372–379. 16291774 10.1093/annonc/mdj057

[6] Ding Q, Luckhardt T, Hecker L, Zhou Y, Liu G, et al (2011) New insights into the pathogenesis and treatment of idiopathic pulmonary fibrosis. Drugs 71: 981–1001. 21668038 10.2165/11591490-000000000-00000 PMC3955181

[7] Liby KT, Sporn MB (2012) Synthetic oleanane triterpenoids: multifunctional drugs with a broad range of applications for prevention and treatment of chronic disease. Pharmacol Rev 64: 972–1003. 22966038 10.1124/pr.111.004846 PMC3462991

[8] Auletta JJ, Alabran JL, Kim BG, Meyer CJ, Letterio JJ (2010) The synthetic triterpenoid, CDDO-Me, modulates the proinflammatory response to in vivo lipopolysaccharide challenge. J Interferon Cytokine Res 30: 497–508. 20626291 10.1089/jir.2009.0100 PMC2950060

[9] Ferguson HE, Kulkarni A, Lehmann GM, Garcia-Bates TM, Thatcher TH, et al (2009) Electrophilic peroxisome proliferator-activated receptor-gamma ligands have potent antifibrotic effects in human lung fibroblasts. Am J Respir Cell Mol Biol 41: 722–730. 19286977 10.1165/rcmb.2009-0006OC PMC2784409

[10] Kulkarni AA, Thatcher TH, Olsen KC, Maggirwar SB, Phipps RP, et al (2011) PPAR-gamma ligands repress TGFbeta-induced myofibroblast differentiation by targeting the PI3K/Akt pathway: implications for therapy of fibrosis. PLoS One 6: e15909. 21253589 10.1371/journal.pone.0015909 PMC3017065

[11] Burgess HA, Daugherty LE, Thatcher TH, Lakatos HF, Ray DM, et al (2005) PPARgamma agonists inhibit TGF-beta induced pulmonary myofibroblast differentiation and collagen production: implications for therapy of lung fibrosis. Am J Physiol Lung Cell Mol Physiol 288: L1146–1153. 15734787 10.1152/ajplung.00383.2004

[12] Thatcher TH, Benson RP, Phipps RP, Sime PJ (2008) High-dose but not low-dose mainstream cigarette smoke suppresses allergic airway inflammation by inhibiting T cell function. Am J Physiol Lung Cell Mol Physiol 295: L412–421. 18567739 10.1152/ajplung.00392.2007 PMC2536795

[13] Lakatos HF, Burgess HA, Thatcher TH, Redonnet MR, Hernady E, et al (2006) Oropharyngeal aspiration of a silica suspension produces a superior model of silicosis in the mouse when compared to intratracheal instillation. Exp Lung Res 32: 181–199. 16908446 10.1080/01902140600817465 PMC10208218

[14] Olsen KC, Sapinoro RE, Kottmann RM, Kulkarni AA, Iismaa SE, et al (2011) Transglutaminase 2 and its role in pulmonary fibrosis. Am J Respir Crit Care Med 184: 699–707. 21700912 10.1164/rccm.201101-0013OC PMC3208598

[15] Wright TW, Gigliotti F, Finkelstein JN, McBride JT, An CL, et al (1999) Immune-mediated inflammation directly impairs pulmonary function, contributing to the pathogenesis of Pneumocystis carinii pneumonia. J Clin Invest 104: 1307–1317. 10545529 10.1172/JCI6688 PMC409816

[16] Bhagwat SP, Wright TW, Gigliotti F (2010) Anti-CD3 antibody decreases inflammation and improves outcome in a murine model of Pneumocystis pneumonia. J Immunol 184: 497–502. 19949093 10.4049/jimmunol.0901864 PMC2797573

[17] Sporn MB, Liby KT, Yore MM, Fu L, Lopchuk JM, et al (2011) New synthetic triterpenoids: potent agents for prevention and treatment of tissue injury caused by inflammatory and oxidative stress. J Nat Prod 74: 537–545. 21309592 10.1021/np100826q PMC3064114

[18] Deeb D, Gao X, Dulchavsky SA, Gautam SC (2008) CDDO-Me inhibits proliferation, induces apoptosis, down-regulates Akt, mTOR, NF-kappaB and NF-kappaB-regulated antiapoptotic and proangiogenic proteins in TRAMP prostate cancer cells. J Exp Ther Oncol 7: 31–39. 18472640

[19] Liby K, Royce DB, Williams CR, Risingsong R, Yore MM, et al (2007) The synthetic triterpenoids CDDO-methyl ester and CDDO-ethyl amide prevent lung cancer induced by vinyl carbamate in A/J mice. Cancer Res 67: 2414–2419. 17363558 10.1158/0008-5472.CAN-06-4534

[20] Konopleva M, Tsao T, Ruvolo P, Stiouf I, Estrov Z, et al (2002) Novel triterpenoid CDDO-Me is a potent inducer of apoptosis and differentiation in acute myelogenous leukemia. Blood 99: 326–335. 11756188 10.1182/blood.v99.1.326

[21] Moeller A, Ask K, Warburton D, Gauldie J, Kolb M (2008) The bleomycin animal model: A useful tool to investigate treatment options for idiopathic pulmonary fibrosis? Int J Biochem Cell Biol 40: 362–382. 17936056 10.1016/j.biocel.2007.08.011 PMC2323681

[22] Saito F, Tasaka S, Inoue K, Miyamoto K, Nakano Y, et al (2008) Role of interleukin-6 in bleomycin-induced lung inflammatory changes in mice. Am J Respir Cell Mol Biol 38: 566–571. 18096870 10.1165/rcmb.2007-0299OC

[23] Kawashima M, yatsunami J, Fukuno Y, Nagata M, Tominaga M, et al (2002) Inhibitory effects of 14-membered ring macrolide antibiotics on bleomycin-induced acute lung injury. Lung 180: 73–89. 12182159 10.1007/pl00021246

[24] Phan SH, Kunkel SL (1992) Lung cytokine production in bleomycin-induced pulmonary fibrosis. Exp Lung Res 18: 29–43. 1374023 10.3109/01902149209020649

[25] Vanoirbeek JA, Rinaldi M, De Vooght V, Haenen S, Bobic S, et al (2010) Noninvasive and invasive pulmonary function in mouse models of obstructive and restrictive respiratory diseases. Am J Respir Cell Mol Biol 42: 96–104. 19346316 10.1165/rcmb.2008-0487OC

[26] Heathcote KL, Cockcroft DW, Fladeland DA, Fenton ME (2011) Normal expiratory flow rate and lung volumes in patients with combined emphysema and interstitial lung disease: a case series and literature review. Can Respir J 18: e73–76. 21969934 10.1155/2011/354325 PMC3267611

[27] Nathan SD, Shlobin OA, Weir N, Ahmad S, Kaldjob JM, et al (2011) Long-term course and prognosis of idiopathic pulmonary fibrosis in the new millennium. Chest 140: 221–229. 21729893 10.1378/chest.10-2572

[28] Jules-Elysee K, White DA (1990) Bleomycin-induced pulmonary toxicity. Clin Chest Med 11: 1–20. 1691067

[29] Kreisman H, Wolkove N (1992) Pulmonary toxicity of antineoplastic therapy. Semin Oncol 19: 508–520. 1411649

[30] Suh N, Wang Y, Honda T, Gribble GW, Dmitrovsky E, et al (1999) A novel synthetic oleanane triterpenoid, 2-cyano-3,12-dioxoolean-1,9-dien-28-oic acid, with potent differentiating, antiproliferative, and anti-inflammatory activity. Cancer Res 59: 336–341. 9927043

[31] Place AE, Suh N, Williams CR, Risingsong R, Honda T, et al (2003) The novel synthetic triterpenoid, CDDO-imidazolide, inhibits inflammatory response and tumor growth in vivo. Clin Cancer Res 9: 2798–2806. 12855660

[32] Honda T, Padegimas EM, David E, Sundararajan C, Liby KT, et al (2010) 2-Cyano-3,10-dioxooleana-1,9(11)-dien-28-oic acid anhydride. A novel and highly potent anti-inflammatory and cytoprotective agent. Bioorg Med Chem Lett 20: 2275–2278. 20188548 10.1016/j.bmcl.2010.02.007 PMC2862379

[33] Hogan CM, Thatcher TH, Sapinoro RE, Gurell MN, Ferguson HE, et al (2011) Electrophilic PPARgamma Ligands Attenuate IL-1beta and Silica-Induced Inflammatory Mediator Production in Human Lung Fibroblasts via a PPARgamma-Independent Mechanism. PPAR Res 2011: 318134. 21765824 10.1155/2011/318134 PMC3135061

[34] Thimmulappa RK, Scollick C, Traore K, Yates M, Trush MA, et al (2006) Nrf2-dependent protection from LPS induced inflammatory response and mortality by CDDO-Imidazolide. Biochem Biophys Res Commun 351: 883–889. 17097057 10.1016/j.bbrc.2006.10.102 PMC2293275

[35] Reddy NM, Suryanaraya V, Yates MS, Kleeberger SR, Hassoun PM, et al (2009) The triterpenoid CDDO-imidazolide confers potent protection against hyperoxic acute lung injury in mice. Am J Respir Crit Care Med 180: 867–874. 19679692 10.1164/rccm.200905-0670OC PMC2773914

[36] Sogno I, Vannini N, Lorusso G, Cammarota R, Noonan DM, et al (2009) Anti-angiogenic activity of a novel class of chemopreventive compounds: oleanic acid terpenoids. Recent Results Cancer Res 181: 209–212. 19213570 10.1007/978-3-540-69297-3_19

[37] Pergola PE, Raskin P, Toto RD, Meyer CJ, Huff JW, et al (2011) Bardoxolone methyl and kidney function in CKD with type 2 diabetes. N Engl J Med 365: 327–336. 21699484 10.1056/NEJMoa1105351

[38] Gauldie J, Bonniaud P, Sime P, Ask K, Kolb M (2007) TGF-beta, Smad3 and the process of progressive fibrosis. Biochem Soc Trans 35: 661–664. 17635115 10.1042/BST0350661

[39] Wynn TA (2008) Cellular and molecular mechanisms of fibrosis. J Pathol 214: 199–210. 18161745 10.1002/path.2277 PMC2693329

[40] Mi S, Li Z, Yang HZ, Liu H, Wang JP, et al (2011) Blocking IL-17A promotes the resolution of pulmonary inflammation and fibrosis via TGF-beta1-dependent and -independent mechanisms. J Immunol 187: 3003–3014. 21841134 10.4049/jimmunol.1004081

[41] Wilson MS, Madala SK, Ramalingam TR, Gochuico BR, Rosas IO, et al (2010) Bleomycin and IL-1beta-mediated pulmonary fibrosis is IL-17A dependent. J Exp Med 207: 535–552. 20176803 10.1084/jem.20092121 PMC2839145

[42] Kolb M, Margetts PJ, Anthony DC, Pitossi F, Gauldie J (2001) Transient expression of IL-1beta induces acute lung injury and chronic repair leading to pulmonary fibrosis. J Clin Invest 107: 1529–1536. 11413160 10.1172/JCI12568 PMC200196

[43] Wang Y, Porter WW, Suh N, Honda T, Gribble GW, et al (2000) A synthetic triterpenoid, 2-cyano-3,12-dioxooleana-1,9-dien-28-oic acid (CDDO), is a ligand for the peroxisome proliferator-activated receptor gamma. Mol Endocrinol 14: 1550–1556. 11043571 10.1210/mend.14.10.0545

[44] Milam JE, Keshamouni VG, Phan SH, Hu B, Gangireddy SR, et al (2008) PPAR-gamma agonists inhibit profibrotic phenotypes in human lung fibroblasts and bleomycin-induced pulmonary fibrosis. Am J Physiol Lung Cell Mol Physiol 294: L891–901. 18162602 10.1152/ajplung.00333.2007 PMC5926773

[45] Kulkarni A, Woeller CF, Thatcher TH, Ramon S, Phipps RP, et al (2012) Emerging PPARγ-Independent Role of PPARγ Ligands in Lung Diseases. PPAR Research 2012: 1–13. 10.1155/2012/705352 PMC3385049 22778711

[46] Catane R, Schwade JG, Turrisi AT 3rd, Webber BL, Muggia FM (1979) Pulmonary toxicity after radiation and bleomycin: a review. Int J Radiat Oncol Biol Phys 5: 1513–1518. 94053 10.1016/0360-3016(79)90761-2

[47] Sime PJ, Xing Z, Graham FL, Csaky KG, Gauldie J (1997) Adenovector-mediated gene transfer of active transforming growth factor-beta1 induces prolonged severe fibrosis in rat lung. J Clin Invest 100: 768–776. 9259574 10.1172/JCI119590 PMC508247

