# Supplementary figures and images for: The Triterpenoid CDDO-Me Inhibits Bleomycin-Induced Lung Inflammation and Fibrosis
Source: PLoS One. 2013 May 31;8(5):e63798. doi: 10.1371/journal.pone.0063798 (PMC3669327; doi:10.1371/journal.pone.0063798)

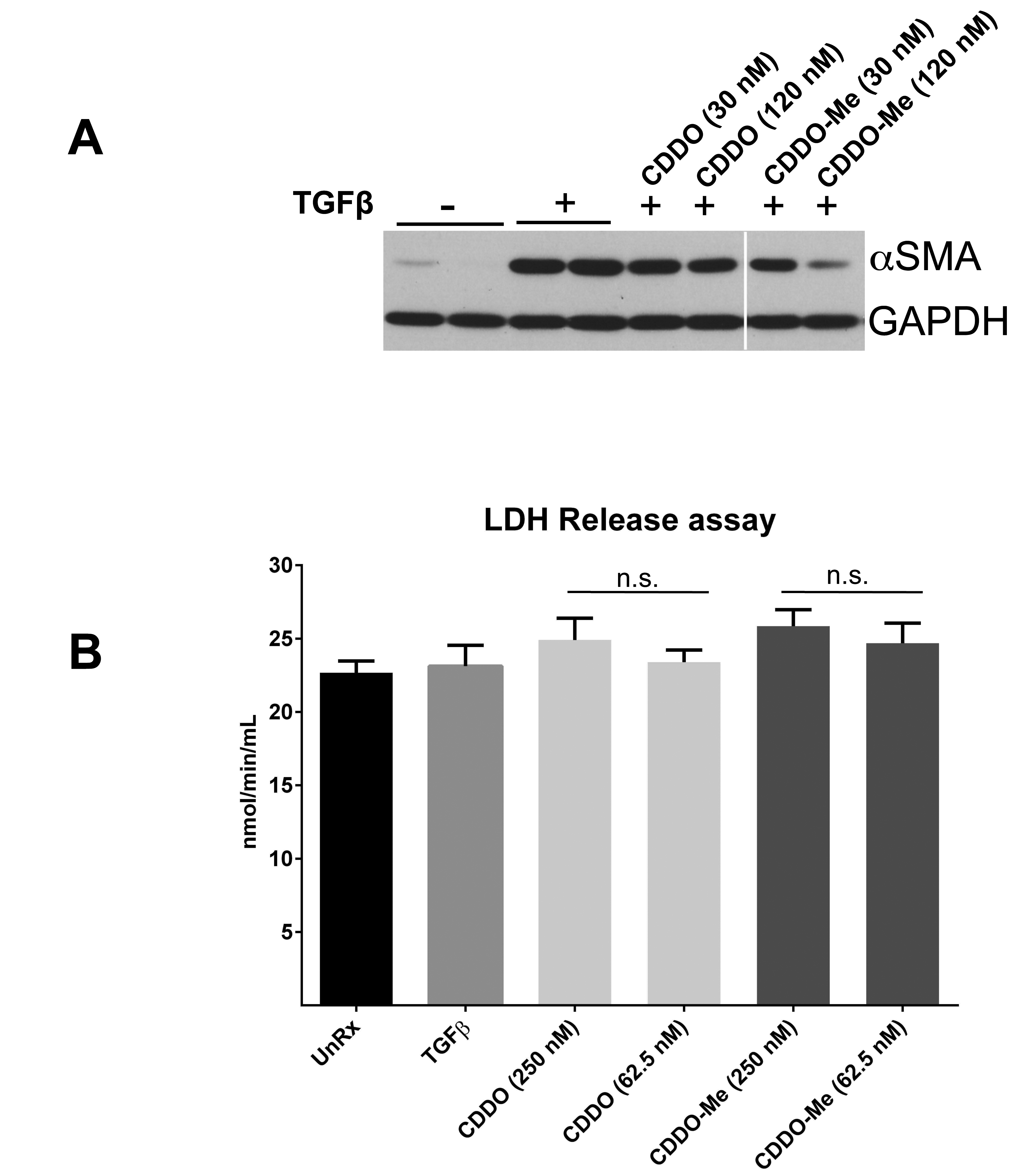

Supplement: Figure S1 — CDDO and CDDO-Me inhibit TGFβ-induced αSMA in a dose dependent manner. (A) Primary HLFs were grown until 70–80% confluent, serum starved for 24 hours and treated with the indicated concentrations of CDDO or CDDO-Me for 48 hours. Total cell lysates were prepared, and subjected to SDS-PAGE followed by immunoblotting. The blot was probed with antibodies against αSMA and loading control GAPDH. (B) Primary HLFs were treated with TGFβ alone, in combination with different doses of either CDDO or CDDO-Me for 72 hours with indicated concentrations or left untreated, and LDH release was measured (nmol/min/mL). (n = 3, mean ± S.E. shown, groups were not significantly (n.s.) different from one another as measured by one way ANOVA). These data indicate that LDH release does not increase in response to either CDDO or CDDO-Me. (TIF) [file pone.0063798.s001.tif]
