# Supplementary material for: The Triterpenoid CDDO-Me Inhibits Bleomycin-Induced Lung Inflammation and Fibrosis
Source: PLoS One. 2013 May 31;8(5):e63798. doi: 10.1371/journal.pone.0063798 (PMC3669327; doi:10.1371/journal.pone.0063798)
Supplement: Table S1 — Preliminary analysis of the four control groups. (DOCX) [file pone.0063798.s002.docx]

Table S1: Preliminary analysis of the four control groups

| **Outcome** | **PBS** | **CDDO-Me (300ng)** | **CDDO-Me (350ng)** | **BLEO** |
| --- | --- | --- | --- | --- |
| Col1A1 mRNA | 1.074, 0.413, 8 | 0.470, 0.086, 4^#^ | 0.940, 0.382, 5 | 5.765, 4.242, 13 * |
| FN mRNA | 1.048, 0.288, 8 | 0.877, 0.152, 4 | 1.191, 0.561, 5 | 10.136, 7.980, 16 * |
| Histology | 0.042, 0.118, 8 | 0.050, 0.112, 5 | 0.233, 0.325, 5 | 2.690, 0.826, 14 * |
| Hydroxy-proline content | 49.638, 10.186, 10 | 49.572, 20.716, 5 | 61.032, 13.241, 5 | 94.44, 19.282, 17 * |
| Compliance | 1.250, 0.149, 10 | 1.185, 0.017, 5 | 1.171, 0.143, 5 | 0.726, 0.243, 17 * |
| Respiratory Rate | 301.619, 49.148, 10 | NA | 307.813, 24.793, 5 | 429.548, 59.976, 16 * |

Summary data for each of the four control groups (mean, standard deviation, sample size). * Indicates significant difference from the PBS group (p-value < 0.05). # Indicates CDDO-Me (300ng) group is significantly different from CDDO-Me (350ng) group (p-value < 0.05).
