# Supplementary material for: The Triterpenoid CDDO-Me Inhibits Bleomycin-Induced Lung Inflammation and Fibrosis
Source: PLoS One. 2013 May 31;8(5):e63798. doi: 10.1371/journal.pone.0063798 (PMC3669327; doi:10.1371/journal.pone.0063798)
Supplement: Table S2 — Tests for the significance of the interaction term in the full model. (DOCX) [file pone.0063798.s003.docx]

Table S2: Tests for the significance of the interaction term in the full model.

| **Outcome** | **F-statistic** | **p-value** |
| --- | --- | --- |
| Col1A1 mRNA | 0.618 | 0.4419 |
| FN mRNA | 0.011 | 0.9163 |
| Histology | 0.117 | 0.7359 |
| Hydroxyproline content | 0.011 | 0.9182 |
| Compliance | 0.077 | 0.7835 |
| Respiratory Rate | 0.306 | 0.5852 |

None of the tests for the interaction term in the full model were significant; therefore the final models did not include the interaction.
