# Supplementary material for: The Triterpenoid CDDO-Me Inhibits Bleomycin-Induced Lung Inflammation and Fibrosis
Source: PLoS One. 2013 May 31;8(5):e63798. doi: 10.1371/journal.pone.0063798 (PMC3669327; doi:10.1371/journal.pone.0063798)
Supplement: Table S3 — Adjusted mean responses for the one treatment strategy and one positive control group. (DOCX) [file pone.0063798.s004.docx]

Table S3: Adjusted mean responses for the one treatment strategy and one positive control group.

| **Outcome** | **Bleomycin** | **Bleo + CDDO-Me** |
| --- | --- | --- |
| Col1A1 mRNA | 5.925 (1.135) | 4.982 (1.393) |
| FN mRNA | 10.725 (1.713) | 5.215 (2.284) |
| Histology | 2.665 (0.192) | 1.997 (0.216) |
| Hydroxyproline content | 93.894 (3.472) | 83.342 (4.014) |
| Compliance | 0.726 (0.055) | 0.932 (0.064) |
| Respiratory Rate | 432.113 (12.423) | 368.007 (13.890) |

Mean responses for the two treatment groups from the model without the interaction after adjusting for CDDO-Me dosage, together with their standard errors (in parentheses).
