# Supplementary material for: The Triterpenoid CDDO-Me Inhibits Bleomycin-Induced Lung Inflammation and Fibrosis
Source: PLoS One. 2013 May 31;8(5):e63798. doi: 10.1371/journal.pone.0063798 (PMC3669327; doi:10.1371/journal.pone.0063798)
Supplement: Table S4 — Test for the treatment strategy versus the positive control. (DOCX) [file pone.0063798.s005.docx]

Table S4: Test for the treatment strategy versus the positive control

| **Outcome** | **CDDO-Me vs Bleo** |
| --- | --- |
| Col1A1 mRNA | 0.522 (0.6079) |
| FN mRNA | 1.892 (0.0717) |
| Histology | 2.309 (0.0307) * |
| Hydroxyproline content | 1.981 (0.0578) |
| Compliance | -2.433 (0.0216) * |
| Respiratory Rate | 3.415 (0.0021) * |

Test statistics using a nominal two-sided t-test calculated for the contrast among the adjusted group means, together with the corresponding p-values (in parentheses). Indicates significant tests (p-value < 0.05).
